# Sex-Related Measurement Bias in Autism Spectrum Disorder Symptoms in the Baby Siblings Research Consortium

**DOI:** 10.1001/jamanetworkopen.2025.25887

**Published:** 2025-08-08

**Authors:** Catherine A. Burrows, Sooyeon Sung, Shuting Zheng, Greg S. Young, Tony Charman, Cheryl Klaiman, Ami Klin, Natasha Marrus, Sally Ozonoff, Joseph Piven, Diana L. Robins, Rebecca J. Schmidt, A. J. Schwichtenberg, Sara Jane Webb, Lonnie Zwaigenbaum, Leslie J. Carver, Katarzyna Chawarska, Suzanne Curtin, Shafali S. Jeste, Jana M. Iverson, Rebecca J. Landa, Daniel S. Messinger, Jane E. Roberts, Wendy L. Stone, Helen Tager-Flusberg, Amy N. Esler, Meghan Miller, Somer L. Bishop, Jed T. Elison

**Affiliations:** 1 Department of Pediatrics, University of Minnesota, Minneapolis; 2 Graduate School of Education, Kyung Hee University, Seoul, Korea; 3 Department of Psychiatry and Behavioral Sciences, University of Texas at Austin, Austin; 4 Department of Psychiatry and Behavioral Sciences, University of California, Davis, Davis; 5 Department of Psychology, King’s College London, London, UK, on behalf of the BASIS Network; 6 Marcus Autism Center, Children’s Healthcare of Atlanta and Emory University School of Medicine, Emory University, Atlanta, Georgia; 7 Department of Psychiatry, Washington University in St Louis, St Louis, Missouri; 8 Department of Psychiatry, University of North Carolina at Chapel Hill, Chapel Hill, on behalf of the IBIS network; 9 A. J. Drexel Autism Institute, Drexel University, Philadelphia, Pennsylvania; 10 School of Medicine, Public Health Sciences, University of California, Davis, Davis; 11 Department of Human Development and Family Science, Purdue University, West Lafayette, Indiana; 12 Department of Psychiatry and Behavioral Sciences, University of Washington, Seattle; 13 Department of Pediatrics, University of Alberta, Edmonton, Alberta, Canada; 14 Department of Psychology, University of California, San Diego, San Diego; 15 Department of Child and Youth Studies, Brock University, St Catharines, Ontario, Canada; 16 Departments of Neurology and Pediatrics, Children’s Hospital Los Angeles, Los Angeles, California; 17 Department of Physical Therapy, Sargent College of Health and Rehabilitation Sciences, Boston University, Boston, Massachusetts; 18 Center for Autism Services, Science and Innovation, Kennedy Krieger Institute, Johns Hopkins University School of Medicine, Baltimore, Maryland; 19 Department of Psychiatry and Behavioral Sciences, Johns Hopkins University School of Medicine, Baltimore, Maryland; 20 Department of Psychology, University of Miami, Miami, Florida; 21 Department of Psychology, University of South Carolina, Columbia; 22 Department of Psychology, University of Washington, Seattle; 23 Department of Psychological and Brain Sciences, Boston University, Boston, Massachusetts; 24 Department of Psychiatry and Behavioral Sciences, University of California, San Francisco, San Francisco; 25 Institute of Child Development, University of Minnesota, Minneapolis

## Abstract

**Importance:**

Disparities exist in age of diagnosis and prevalence of autism spectrum disorder (ASD) for female compared with male children. Correcting for sources of bias is critical for improving equitable ASD identification.

**Objective:**

To determine whether sex differences exist in measurement of ASD symptoms using the Autism Diagnostic Observation Schedule (ADOS) among young children at high familial likelihood (HFL) and low familial likelihood (LFL) of ASD.

**Design, Setting, and Participants:**

This cohort study collected longitudinal, prospective data from the Baby Siblings Research Consortium between January 1, 2003, and December 31, 2021. Participants included 3106 children who had an older sibling with ASD (HFL group) and 1444 without (LFL group). Data from as many as 3 visits when participants were aged 20 to 40 months were included. Analysis occurred between March 1, 2023, and May 29, 2025.

**Exposures:**

Child sex and age and ASD diagnosis.

**Main Outcomes and Measures:**

Measurement invariance by sex and age was examined across item-level ADOS data. Diagnostic group and sex differences were then examined using mixed-effect models on corrected scores.

**Results:**

Repeated visits (n = 7557) from 4550 participants (2548 [56.0%] male) were included, of whom 1444 (31.7%) were in the LFL and 3016 (68.3%) in the HFL groups. Confirmatory factor analysis indicated social communication and restricted and repetitive behaviors models fit the data well in the HFL group but poorly in the LFL group. In the HFL group, females were rated as less impaired in eye contact (differential item functioning estimate [SE] = 0.088 [0.033]; *P* = .01), and their response to joint attention (differential item functioning estimate [SE] = 0.290 [0.105]; *P* = .01) and quality of social overtures (differential item functioning estimate [SE] = 0.053 [0.019]; *P* = .005) was associated with less underlying social communication difficulties compared with males. Adjusting for differential item functioning by age and sex resulted in moderate levels of measurement differences. Females showed milder autistic traits than males, although this gap was smaller in the participants diagnosed with ASD.

**Conclusions and Relevance:**

Sex differences exist in the general population in many social communication traits, yet ASD diagnostic thresholds do not account for these sex differences. Future instrument development, as well as clinician training, should acknowledge milder presentation (fewer difficulties with eye contact or quality of social impairments) in many females. This may help identify developmental differences earlier and improve outcomes for autistic females (estimate [SE] = −0.160 [0.061]; *P* = .009).

## Key Points

**Question:**

Do sex differences exist in the structure and level of autism symptoms in prospectively ascertained young children at high and low familial likelihood for autism spectrum disorder (ASD)?

**Findings:**

In this cohort study of 4550 participants, an ASD diagnostic instrument demonstrated moderate measurement differences between sexes, and females showed milder autistic traits than males, although this gap was smaller in the participants diagnosed with ASD. ASD diagnostic thresholds did not account for sex differences in the general population.

**Meaning:**

These findings suggest that future instrument development and clinician training should acknowledge milder presentation in many females to identify developmental differences earlier and improve outcomes for females with ASD symptoms.

## Teaser

This cohort study evaluates sex- and age-related measurement bias on the Autism Diagnostic Observation Schedule in a large, pooled sample of infant siblings at high and low familial likelihood of autism spectrum disorder.

## Introduction

While autism spectrum disorder (ASD) can be reliably diagnosed in early childhood, females are often diagnosed later than males.^1,2,3,4^ Our understanding of ASD is based on predominantly male samples, consistent with the 4:1 sex ratio in epidemiological studies.^5,6,7^ Few sex differences have been found in symptom levels, though autistic females tend to show milder restricted and repetitive behaviors (RRBs) compared with autistic males.^1,8,9,10^ Nearly all studies examining the structure of autism symptoms use samples of children who were referred for an ASD evaluation.^11,12,13,14^ Autistic females are more likely to be missed than males in early development,^2,6,15^ and thus are underrepresented in analyses of sex differences. It is unclear whether females are missed in early development due to biases within screening and referral processes,^16,17,18^ biases in clinician decision-making,^7,19,20,21^ measurement differences,^14,22^ or true sex differences in the emergence of early signs and symptoms of ASD.^22,23^ Investigating measurement properties of early ASD assessment measures and leveraging a putatively less biased ascertainment strategy could elucidate why fewer females are identified early in development.

Prospective studies of infant siblings of children with ASD avoid sex biases in referral processes, as children are recruited in infancy independent of clinical concern. Studies prospectively monitoring for ASD in samples of children at high familial likelihood (HFL) have found lower sex ratios, closer to 2:1.^4,24,25^ Within HFL sibling samples, a wide range of ASD symptoms are present. Approximately 20% of HFL siblings meet criteria for ASD,^26,27^ and an additional 20% to 30% show subclinical features.^28,29,30^ Male HFL siblings without ASD show higher autism symptoms and lower developmental levels compared with female HFL siblings without ASD.^1,28^ When correcting for measurement bias, females demonstrate milder social communication (SC) and RRB symptoms than males across infancy and toddlerhood, regardless of diagnosis.^22^

The Autism Diagnostic Observation Schedule (ADOS) is a well-validated observational tool designed to capture SC impairments and RRBs.^31,32^ It was originally developed to augment clinical judgment in the context of autism diagnostic assessment, and a large body of literature supports its general effectiveness in differentiating autistic individuals from nonspectrum clinical referrals.^33,34^ A subset of behaviors scored during the ADOS is included on a diagnostic algorithm that generates both SC and RRB severity scores.

When scores are combined from a subset of items on an instrument, the new summed total inherently takes on the attributes of a latent construct. ADOS severity scores were developed as a psychometrically sound method of measuring and comparing core features of autism across samples, ages, and language levels over time in research and clinical assessment.^35^ However, subsequent research has applied these scores to other groups (eg, HFL siblings) despite a lack of psychometric scrutiny to demonstrate the validity of the ADOS severity scores in measuring ASD traits in nonautistic populations. To use the ADOS to index meaningful variability in SC and RRBs, it is imperative to establish construct validity in the samples of interest.^36^

This cohort study evaluates sex- and age-related measurement bias on the ADOS in a large, pooled sample of infant HFL siblings and those at low familial likelihood (LFL) for ASD at 20 to 40 months of age. We examined sex differences in the underlying factor structure of SC and RRB to determine the magnitude of sex-related measurement bias. We then evaluated ASD diagnostic group and sex-related differences in scores adjusted for sex-related measurement bias. We expected to find differences between children with and without autism, as well as small sex differences in SC and RRB scores.

## Methods

### Participants

Participants were drawn from the Baby Siblings Research Consortium (BSRC) database and included younger siblings of children with ASD (HFL group) and a comparison sample of children who did not have an older sibling with ASD (LFL group) who were seen for visits between January 1, 2003, and December 31, 2021. Included participants had ADOS data collected between 20 and 40 months of age. Clinical best estimate (CBE) diagnoses of ASD (ASD-positive vs ASD-negative) were completed by sites; the CBE at the latest visit was used to determine ASD diagnosis. Because race and ethnicity were reported differently for each BSRC site, we collapsed categories into White or other race (including American Indian or Alaska Native, Asian, Black, Native Hawaiian or Other Pacific Islander, multiracial, and other) and Hispanic or non-Hispanic ethnicity, with additional sample characterization in eTables 1 and 2 in Supplement 1.

### Procedures

Data were obtained from the BSRC database (18 sites [eTable 3 in Supplement 1]) in December 2022. Institutional review board approval and written informed consent for all participants were obtained at each study site. We followed the Strengthening the Reporting of Observational Studies in Epidemiology (STROBE) reporting guideline.

### Measures

The ADOS, Generic (ADOS-G)^32^ and 2nd Edition (ADOS-2)^31^ versions, is a semistructured diagnostic observation with an examiner that was developed to identify the presence of autism-related behaviors and symptoms. Behaviors are scored on a 4-point scale, with higher scores more indicative of symptoms in the area assessed. We selected SC and RRB algorithm items that are rated in Modules Toddler, 1, and 2 for both ADOS-G and ADOS-2 (Box). Immediate echolalia was also included in the RRB factor, given its relevance to the domain^13^ and inclusion in past factor analysis studies.^13,16^ Expressive language level was derived by remapping scores from each ADOS module onto a uniform scale following Mazurek et al,^37^ wherein higher scores are indicative of more advanced language skills. Calibrated Severity Scores were also calculated for use in the mixed-model analyses.^38^

Box. **ADOS Items Included for Social Communication and Restricted and Repetitive Behavior Domains**

**Social Communication**

Unusual eye contact
Facial expressions directed to others
Showing
Shared enjoyment in interaction
Spontaneous initiation of joint attention
Gestures
Pointing
Response to name
Response to joint attention
Quality of social overtures

**Restricted and Repetitive Behavior**

Hand and finger and other complex mannerisms
Immediate echolalia
Intonation of vocalizations and/or verbalizations
Stereotyped or idiosyncratic use of words and/or phrases
Unusually repetitive interests and/or stereotyped behavior
Unusual sensory interest in play material or person

### Statistical Analysis

#### Moderated Nonlinear Factor Analysis

Analysis occurred between March 1, 2023, and May 29, 2025. Moderated nonlinear factor analysis (MNLFA) simultaneously characterizes the extent of sex- and age-related measurement bias using continuous and categorical moderators and generates estimates of underlying SC and RRB.^39^ MNLFA has advantages compared with traditional factor analysis and item response theory, including its flexibility allowing examination of multiple, simultaneous moderators, either continuous or categorical and complex patterns of differential item functioning (DIF) without strong assumptions. We evaluated for intercept- and loading-level DIF on moderators of interest: sex (dummy coded as male = 1 and female = 0), age (modeled continuously in months to 2 decimal places), and likelihood group (dummy coded as HFL = 1 and LFL = 0) on factors of SC and RRB.^39^ Intercept-level DIF reflects different mean levels of the indicator, while loading-level DIF represents a different association between the indicator and underlying latent construct, by moderator. The effects of moderators on the latent factor and variance were also examined. We first evaluated configural invariance at each level of the moderators. We then drew 5 different calibration samples, given the high number of participants with longitudinal data. The model parameter estimation within each calibration sample was conducted through 3 steps: (1) effect of moderators on DIF for intercept and loading was examined for each indicator separately; (2) all effects with *P* < .05 from the previous step were tested simultaneously in a single model; and (3) the final model was estimated using the significant parameters (*P* < .05) after the Benjamini-Hochberg correction from the previous step. Final parameter estimates were then combined from the 5 calibration samples based on Rubin’s rules^40^ to produce the final factor score estimation for each participant at each visit. To interpret effects across the 5 calibration samples, pooled estimates using a conservative *P* ≤ .01 threshold are discussed given the high number of effects tested. Due to potential differences across ADOS modules, MNLFA models were separately run within each module, correcting for sex-related measurement bias (eTable 8 in Supplement 1).

The root expected mean square difference (REMSD)^12^ evaluated the effect size of the difference between the MNLFA-derived DIF-adjusted factor scores (FS MNLFA) and factor scores assuming full measurement invariance without adjusting for DIF (FS FI). It was calculated with the SD of the latent factor score (σ_FS_) fixed to 1 with the following equation:

REMSD = √E [(FS_MNLFA_ – FS_FI_)^2^]/ σ_FS_

#### Mixed-Effects Model

To characterize sex differences in SC and RRB over time, we tested the effects of sex, age, language level, and ASD diagnostic status on the FS MNLFA using a mixed-effects model, including repeated measures within individuals. A series of models were fitted sequentially including (1) random intercept and random slope for individuals and sites, (2) fixed effects of factors associated with outcomes, and (3) interactions between them. Models were compared using χ^2^ log-likelihood ratio tests and second-order Akaike information criteria (AIC). Nonsignificant random and interaction effects were dropped from the final model. All models were fit using the nlme package, version 3.1-164, in R (R Program for Statistical Computing). Age was not centered, but we reran models with age centered at 30 months to avoid potential impacts of collinearity.

## Results

### Sex Ratio

The final sample size included 3106 HFL participants (1330 [42.8%] female and 1776 [57.2%] male) and 1444 LFL participants (672 [46.5%] female and 772 [53.5%] male), totaling 4550 participants (2002 female and 2548 male; 2432 [53.5%] White and 2079 [45.7%] other race; 376 [8.3%] Hispanic and 4035 [88.7%] non-Hispanic ethnicity). Participants had 1 to 3 visits in our age range, resulting in 5298 visits for HFL participants and 2259 visits for LFL participants. Sample information is available in Table 1 and the eMethods in Supplement 1. Within our sample of 2339 HFL participants with ASD diagnostic information, 339 of 1279 males (26.5%) males and 137 of 1022 females (13.4%) females received ASD diagnoses, resulting in an ASD sex ratio of 1.98:1.

**Table 1.** Demographic Statistics for Sample Grouped by Familial Likelihood Status

Characteristic	Participant group		*P* value
	LFL (n = 1444)	HFL (n = 3106)	
Sex, No. (%)			
Female	672 (46.5)	1330 (42.8)	.02^a^
Male	772 (53.5)	1776 (57.2)	
Race, No. (%)			
White	745 (51.6)	1687 (54.3)	.09^a^
Other	687 (47.6)	1392 (44.8)	
Missing	12 (0.8)	27 (0.9)	
Ethnicity, No. (%)			
Hispanic	91 (6.3)	285 (9.2)	.001^a^
Non-Hispanic	1322 (91.6)	2713 (87.3)	
Missing	1 (0.1)	108 (3.5)	
ASD diagnosis, No. (%)			
Positive	0	486 (15.6)	<.001^b^
Negative	1010 (69.9)	1853 (59.7)	
Missing	434 (30.1)	767 (24.7)	
Age at visit			
20-26 mo			
No. of visits	1157	2431	NA
Mean (SD), mo	24.23 (0.81)	24.18 (0.91)	.11^c^
27-35 mo			
No. of visits	199	704	NA
Mean (SD), mo	28.33 (2.37)	28.64 (2.29)	.09^c^
36-40 mo			
No. of visits	903	2163	NA
Mean (SD), mo	36.77 (1.06)	36.89 (1.14)	.007^c^
Age at latest CBE, mean (SD) [range], mo	34.54 (4.92) [21.06-40.48]	33.99 (5.18) [20.96-40.48]	<.001^c^

Abbreviations: ASD, autism spectrum disorder; CBE, clinical best estimate; HFL, high familial likelihood; LFL, low familial likelihood; NA, not applicable.

^a^ Calculated using Pearson χ^2^ test.

^b^ Calculated using Fisher exact test.

^c^ Calculated using independent sample *t* test.

### Aim 1: Measurement Bias by Sex and Age

For HFL participants, the unidimensional SC and RRB confirmatory factor analyses (CFAs) demonstrated good fit for each combination of sex and age bins (eResults and eTable 5 in Supplement 1). Additional models showed adequate fit for nonautistic HFL participants (eTable 6 in Supplement 1). For LFL participants, the unidimensional structure of SC demonstrated poor fit in all age groups for both sexes. The unidimensional model of RRB demonstrated acceptable fit in all sex and age groups except for females aged 27 to 35 months. We did not further investigate measurement invariance in LFL participants for either SC or RRB due to poor SC model fit.

In HFL participants (Table 2), response to joint attention and quality of social overtures exhibited loading DIF by sex (response to joint attention estimate [SE] = 0.290 [0.105], *P* = .01; quality of social overtures estimate [SE] = 0.053 [0.019], *P* = .005); they were stronger indicators of latent SC for males than for females. Showing (estimate [SE] = −0.007 [0.002]; *P* = .003), shared enjoyment in interaction (estimate [SE] = −0.005 [0.001]; *P* = .004), and response to name (estimate [SE] = −0.010 [0.002]; *P* < .001) exhibited loading DIF by age; children received lower rating (ie, fewer symptoms) on these items as they aged while holding levels of latent SC constant. Gestures (estimate [SE] = 0.007 [0.002]; *P* = .004) exhibited intercept DIF by age; children were rated higher (ie, more symptoms) on this item as they aged while holding levels of latent SC constant. Unusual eye contact demonstrated intercept-level DIF by sex (estimate [SE] = 0.088 [0.033]; *P* = .01); males had higher ratings (ie, more difficulties) than females, while holding levels of latent SC constant.

**Table 2.** Moderated Nonlinear Factor Analysis Final Model Parameter Estimation Results for Social Communication and Restricted and Repetitive Behaviors Domains

Item	Source	Parameter estimate (SE)^a^	*P* value^b^
**Social communication**			
Loading			
Unusual eye contact	NA	0.601 (0.017)	NA
Facial expressions directed to others	NA	0.479^c^^,^^d^	NA
Showing	NA	0.424 (0.015)	NA
Shared enjoyment in interaction	NA	0.308 (0.009)	NA
Spontaneous initiation of joint attention	NA	0.401 (0.017)	NA
Gestures	NA	0.236 (0.015)	NA
Pointing	NA	0.387 (0.014)	NA
Response to name	NA	0.352 (0.017)	NA
Response to joint attention	NA	0.229^c^	NA
Quality of social overtures	NA	0.359^c^	NA
Intercept			
Unusual eye contact	NA	0.366^c^	NA
Facial expressions directed to others	NA	0.351 (0.052)	NA
Showing	NA	0.764^c^	NA
Shared enjoyment in interaction	NA	0.324^c^	NA
Spontaneous initiation of joint attention	NA	0.379 (0.051)	NA
Gestures	NA	0.349^c^	NA
Pointing	NA	0.506 (0.047)	NA
Response to name	NA	0.678^c^	NA
Response to joint attention	NA	0.117 (0.030)	NA
Quality of social overtures	NA	0.245^c^	NA
Loading DIF			
Response to joint attention	Sex	0.290 (0.105)	.01
Quality of social overtures	Sex	0.053 (0.019)	.005
Intercept DIF			
Unusual eye contact	Sex	0.088 (0.033)	.01
Showing	Age	−0.007 (0.002)	.003
Shared enjoyment in interaction	Age	−0.005 (0.001)	.004
Gestures	Age	0.007 (0.002)	.004
Response to name	Age	−0.010 (0.002)	<.001
Mean impact η	Sex	0.930 (0.190)	<.001
	Sex × age	−0.010 (0.007)	.005
Variance impact η	NA	1.000 (NA)	NA
**Restricted and repetitive behaviors**			
Loading			
Hand and finger and other complex mannerisms	NA	0.344 (0.015)	NA
Immediate echolalia	NA	−0.053^c^	NA
Intonation of vocalizations/verbalizations	NA	0.451 (0.016)	NA
Stereotyped/idiosyncratic use of words/phrases	NA	0.383 (0.018)	NA
Unusually repetitive interests/stereotyped behavior	NA	0.391 (0.015)	NA
Unusual sensory interest in play material or person	NA	0.246^c^	NA
Intercept			
Hand and finger and other complex mannerisms	NA	0.357 (0.082)	NA
Immediate echolalia	NA	0.590 (0.086)	NA
Intonation of vocalizations or verbalizations	NA	0.154^c^	NA
Stereotyped or idiosyncratic use of words and/or phrases	NA	0.263 (0.093)	NA
Unusually repetitive interests and/or stereotyped behavior	NA	0.692^c^	NA
Unusual sensory interest in play material or person	NA	0.218 (0.063)	NA
Loading DIF			
Immediate echolalia	Age	0.011 (0.003)	.005
Unusual sensory interest in play material or person	Sex	0.109 (0.026)	<.001
Intercept DIF			
Unusually repetitive interests or stereotyped behavior	Age	−0.010 (0.003)	.002
Mean impact η	NA	0.000 (NA)	NA
Variance impact η	NA	1.000 (NA)	NA

Abbreviation: DIF, differential item functioning; NA, not applicable.

^a^ Combined parameter estimates and pooled SE were computed based on Rubin rules.^40^

^b^ Indicates whether the estimate is significantly different from zero.

^c^ Presents intercept in the equation including the effects of moderators. For the full equation see eTable 10 in Supplement 1.

For RRB, unusual sensory interest exhibited loading DIF by sex (estimate [SE] = 0.109 [0.026]; *P* < .001); it was a stronger indicator of the latent RRB factor for males. Immediate echolalia exhibited loading DIF by age (estimate [SE] = 0.011 [0.003]; P = .005); it was a stronger indicator of latent RRB for older children. For intercept DIF by age, unusually repetitive interests or stereotyped behavior had a negative value (estimate [SE] = −0.010 [0.003]; *P* = .002), indicating that children had lower scores as they aged while holding levels of latent RRB constant.

Adjusting item-level DIF had moderate effects on the estimated SC-MNLFA and RRB-MNLFA factors (REMSD-SC = 0.42 and REMSD-RRB = 0.41). There was larger magnitude of measurement bias for sex compared with age for SC (REMSD-SC for sex = 0.41; REMSD-SC for age = 0.01), while age-related measurement bias was larger than sex for RRBs (REMSD-RRB for sex = 0.07; REMSD-RRB for age = 0.34).

### Aim 2: Mixed-Effects Model

Mixed-effects model results are presented in the Figure and Table 3. Results were the same when age was centered at 30 months. For both models, random intercept and linear slope effects were significant for individuals and sites (eTable 11 in Supplement 1), indicating that there were noteworthy individual differences in children’s intercept and rates of change around mean trajectory. For SC, children with ASD (estimate [SE], 1.528 [0.126]) and those with lower language levels (estimate [SE], −0.188 [0.013]) had higher scores (more SC difficulties). Interaction effects indicated that the difference between children with and without ASD was larger at later ages (estimate [SE], 0.024 [0.005]) and that diagnostic differences were smaller at higher language levels (estimate [SE], −0.210 [0.021]). The sex by ASD diagnosis interaction was significant, indicating that the SC differences between ASD-positive and ASD-negative groups were greater for females.

**Figure.**
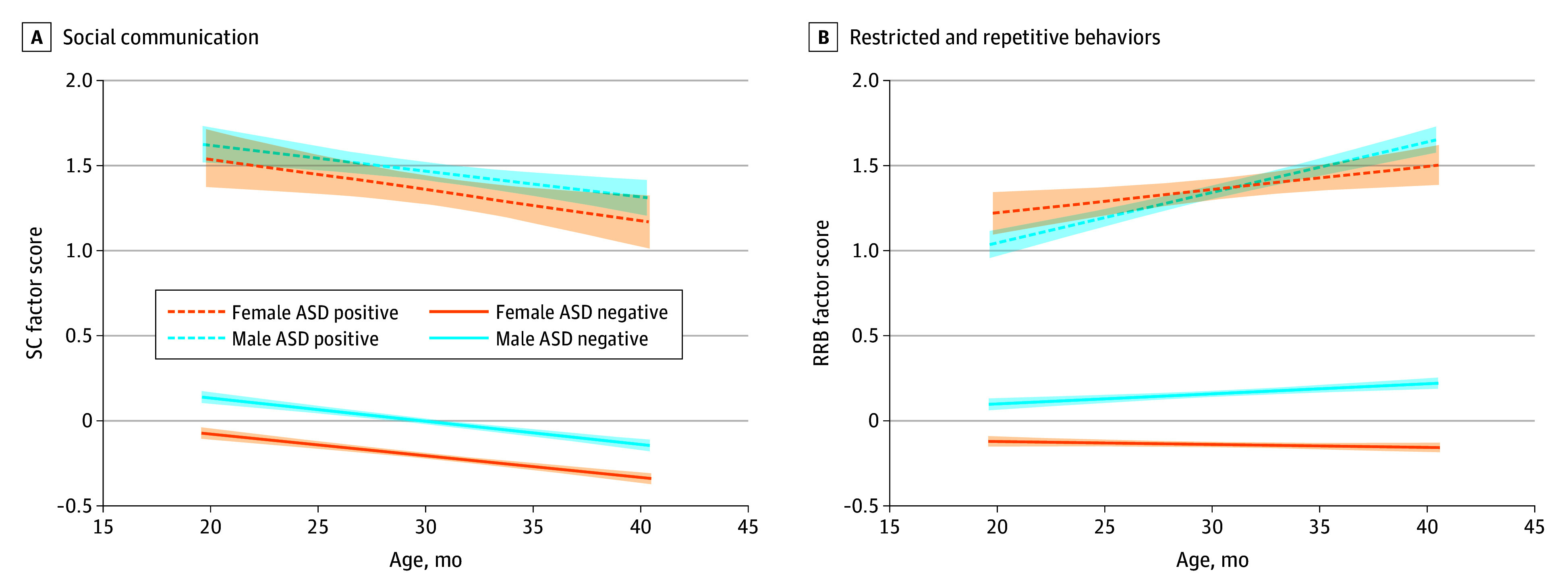
Visual Representation of the Trajectory of Social Communication (SC) and Restricted and Repetitive Behaviors (RRBs) Development Over Time Symptom development is stratified by sex and autism spectrum disorder (ASD) diagnosis. Shading indicates 95% CIs.

**Table 3.** Mixed-Effects Models Associated With Moderated Nonlinear Factor Analysis–Adjusted Social Communication and Restricted and Repetitive Behaviors Scores^a^

Domain and model	Estimate (SE)	*t* Value	*P* value
**Social communication fixed effects**			
(Intercept)	0.579 (0.228)	2.535	.01
Age	−0.004 (0.008)	−0.446	.66
Sex	0.064 (0.094)	0.685	.49
ASD diagnosis	1.528 (0.126)	12.136	<.001
Language level	−0.188 (0.013)	−4.357	<.001
Age × sex	0.003 (0.004)	0.874	.38
Age × ASD diagnosis	0.024 (0.005)	5.199	<.001
Age × language level	0.001 (0.001)	1.064	.29
Sex × ASD diagnosis	−0.160 (0.061)	−2.617	.009
Sex × language level	−0.001 (0.020)	−0.035	.97
ASD diagnosis × language level	−0.210 (0.021)	−9.992	<.001
**Restricted and repetitive behaviors fixed effects**			
(Intercept)	−0.398 (0.244)	−1.632	.10
Age	0.017 (0.009)	1.922	.055
Sex	0.047 (0.096)	0.483	.63
ASD diagnosis	0.842 (0.130)	6.480	<.001
Language level	0.010 (0.045)	0.216	.83
Age × sex	0.016 (0.004)	3.932	<.001
Age × ASD diagnosis	0.024 (0.005)	4.775	<.001
Age × language level	−0.002 (0.001)	−1.485	.14
Sex × ASD diagnosis	−0.367 (0.066)	−5.543	<.001
Sex × language level	−0.052 (0.022)	−2.415	.02
ASD diagnosis × language level	−0.034 (0.023)	−1.517	.13

Abbreviation: ASD, autism spectrum disorder.

^a^ Variables associated with outcomes examined included age, sex, ASD diagnosis, and language level (assessed on the Autism Diagnostic Observation Schedule), as well as their interactions. Random intercepts were included for site and individuals. Full information is provided in eTable 11 in Supplement 1. Best-fitting models are reported here.

For RRB, autistic children had higher RRB scores (estimate [SE], 0.842 [0.130]). Sex, age, and language levels were not associated with RRB. Interaction effects indicated that increases in RRBs over time were greater in children with ASD (estimate [SE], 0.024 [0.005]) and in males (estimate [SE], −0.367 [0.066]). A sex by language level interaction (estimate [SE], −0.052 [0.022]) indicated that males had a stronger negative association between language level and RRB. Similar to SC, diagnostic differences were larger for females.

## Discussion

This is the largest study to date, to our knowledge, that examines sex differences in ASD symptoms among HFL siblings followed up prospectively. We found that HFL males were 2 times more likely to be diagnosed with ASD than HFL females, which is lower than the sex ratio observed in epidemiological studies^6,40^ and universal screening studies.^25^ We also found sex-related differences in structure and degree of ASD symptoms when correcting for measurement bias. A clinician’s ratings of eye contact, response to joint attention, and the quality of the child’s overtures function differently for males and females. No indicator performed better for females compared with males, indicating a need to develop measures that robustly capture relevant ASD symptoms in young females. The difference in both SC and RRB traits between HFL children with and without ASD was larger for females, highlighting that females need to demonstrate a larger deviation from the average female level of SC difficulties than do males to receive an ASD diagnosis. Sex differences exist in SC and RRBs in the general population but are rarely considered as part of ASD diagnostic processes. Future instrument development, as well as clinician training, should acknowledge milder presentation (ie, fewer difficulties with eye contact or quality of social impairments) in many females.

While the overall factor structure of SC and RRB was similar for males and females, we observed several item-level differences by sex and age. There was larger measurement bias arising from sex relative to age for our investigation of SC. For SC, females exhibited less difficulty with eye contact than males, given comparable SC difficulties. Social overtures and responding to joint attention were less strongly related to underlying SC for females than males. A relative strength in eye contact for females could affect other qualitative codes and lead to less clinician-rated impairment in overtures due to perceived sociality.^41,42^ Future research is needed to identify traits that index SC well for females; it may be that females show strong eye contact but have difficulty coordinating with other forms of communication. Several measurement differences by age were also noted, highlighting the benefits of adjusting the ADOS module and codes based on the child’s language level and age.^38^

Sex differences exist in the general population for many ASD traits, including social motivation,^43^ social reciprocity,^23^ language skills,^44^ and RRBs.^1,45^ However, ASD diagnostic thresholds do not consider these sex differences in ASD traits. Thus, females must show a greater difference from their sex-normed social behaviors and RRBs to meet criteria for ASD, which is consistent with our results of smaller diagnostic group differences in SC and RRBs for males. Failing to account for these sex differences in the general population has reinforced historical biases conferred by considering ASD as a male-dominant disorder.^46^ It could also be that females with milder presentations demonstrate nuanced symptoms that are not captured in the ADOS.^47^ ASD diagnostic measures and criteria may benefit from considering sex differences in behaviors, as the same level of ASD symptoms may carry different implications for males and females.

In the RRB domain, there were greater measurement differences due to age, relative to sex. Sensory interests were more strongly associated with RRBs for among males, while echolalia was more strongly associated with RRBs among older children. Children also showed lower repetitive interests as they grew older, given comparable underlying RRB levels. This is consistent with past reports that have found more age-related DIF in higher order RRBs compared with repetitive motor mannerisms or in self-directed behavior.^45^

Our study examined measurement bias by sex on the ADOS. We only examined 1 measure of ASD symptoms; thus, it is unclear whether our findings of sex bias reflect sex differences in measurement of features of ASD generally or only specifically as measured by the ADOS. Clinical practice should not rely on only 1 measure to inform a diagnosis.^48^ Conducting similar psychometric analyses across multiple measures of SC and RRBs and in different modalities (ie, observational measures, clinical interviews, parent questionnaires) may help to determine whether certain measures are more prone to bias and where differences in measures reflect true sex differences in symptoms. It will be important to stratify these analyses by age, language level, and age of diagnosis, when applicable.

According to our psychometric assessment, the ADOS does not capture meaningful variability in SC or RRBs in the LFL group. Different measures are needed to capture the fine-grained differences in SC and RRBs over time across a range of ages and ability levels and across clinical groups.^49,50^ The ADOS was developed to categorically (not dimensionally) differentiate ASD from non-ASD cases and therefore is not equipped to capture meaningful variability in symptoms outside a clinical diagnostic context.^31,32^ Thus, while the ADOS may be appropriate for ruling out ASD in LFL groups, there is no psychometric support for using scores in dimensional analyses. Although questionnaires exist that capture quantitative variability in SC and RRBs, future work should develop observational measures that capture dimensional variability across atypical and typical development and scrutinize the psychometric integrity of the ADOS in other clinical groups at higher likelihood for ASD (eg, consecutive clinical referrals, genetic conditions, or deaf children).

### Limitations

This study has some limitations. We leveraged a large multisite database to conduct, to our knowledge, the largest investigation of sex differences in HFL siblings to date. However, pooling data across multiple studies introduces variability in diagnostic procedures and thresholds for assigning an ASD diagnosis. Data were also collected across a timeframe where ASD diagnostic criteria changed according to editions of the *Diagnostic and Statistical Manual of Mental Disorders* (from the *DSM-IV* to *DSM-5*). The sex ratio (male to female) for ASD has also changed over time, both in infant sibling studies (2.8:1 in 2011 compared to 2:1 in 2024)^26,27^ and in the general population in the US (from 4.5:1 in 2006 to 3.4:1 in 2022).^40,51^ Thus, cohort effects may exist in our sample that spans a wide data collection range. Date of visit and *DSM* version used for CBE are not included in the BSRC database; thus, it is impossible to probe these limitations further. We also did not control for site as a moderator in the MNLFA analyses due to a high number of sites and small sample sizes in some sites.

Because the ADOS is used as a tool to augment clinical judgment, scores on this measure can never be fully independent from diagnostic determination. There is also no ground truth of whether an individual has ASD as there is for other medical conditions. Thus, the diagnostic group differences in the ADOS are inherently tied to ADOS scores, as several sites required that children met the diagnostic threshold on the ADOS to receive a diagnosis. This is not in line with the Standards for Reporting of Diagnostic Accuracy Studies guidelines,^52^ although using the ADOS as part of a comprehensive clinical evaluation is considered best practice.^48^ It remains important to characterize the effects of sex-related measurement bias to understand how the ADOS functions across different groups.

We also treated ASD as a person-level variable. When assessed multiple times between 18 and 36 months of age, many children will receive different ASD diagnostic outcomes, with 40% to 60% of those meeting ASD criteria at 36 months of age showing subthreshold symptoms at earlier ages.^34,35^ Females may also be more likely to change diagnoses during development,^53,54,55,56^ which could affect diagnostic group comparisons over time in this study. Several variables of interest to our study also covary, including age and ADOS module, with younger participants more likely to receive Module 1 or the Toddler Module. Slight differences in coding anchors could affect our MNLFA analyses. We also found higher language levels for females across ages and diagnostic groups, resulting in more females receiving a Module 2, regardless of ASD diagnosis. It is difficult to tease apart the effects of age, module, language level, and sex. Past research has also shown measurement differences by race and ethnicity.^14^ Examinations of measurement bias by race and ethnicity were beyond the scope of this report, particularly given site-related differences in characterization of race and ethnicity, but future large-scale investigations should test whether there are structural differences in measurement of ASD symptoms by race and ethnicity in HFL samples.

## Conclusions

In this cohort study of HFL siblings, we corrected for sex-related bias to minimize several sources of bias in evaluating sex differences in ASD. The ADOS captured meaningful variability in SC and RRB for children at HFL for ASD, although we identified moderate sex- and age-related measurement bias. Females showed less impairment in eye contact, which is one of the most prominent ASD symptoms; this could help explain why identification of females with ASD may be missed in early childhood. Future work is needed to better understand whether these sources of sex-related measurement bias reflect sex differences in ASD traits or bias in ratings on this measure. Different measures are also needed that capture SC and RRBs meaningfully across various language levels, ages, and the ASD diagnostic continuum. When we correct for sex-related measurement bias, females show milder SC difficulties regardless of diagnosis, highlighting potential avenues to improve identification of ASD. Primary care clinician training, with an eye to milder presentations in females that may include fewer difficulties with eye contact or differences in the quality of social impairments, may help identify concerns earlier and improve outcomes for autistic females.

## Notes

Supplement 1. **eMethods.** Participants, Measures, and Analytic Approaches **eResults.** MNLFA Results **eTable 1.** Demographic Characterization of Sample **eTable 2.** Mullen Early Learning Composite (ELC) Scores by Group **eTable 3.** Baby Sibling Research Consortium (BSRC) Site List With Principal Investigator (PI) **eTable 4.** ADOS Version and Module Administered **eTable 5.** Multigroup Confirmatory Factor Analysis (CFA) Configural Invariance by Sex and Age for HFL and LFL Participants **eTable 6.** Confirmatory Factor Analysis (CFA) Results for HFL-Negative Participants **eTable 7.** Descriptive Statistics of the MNLFA-Derived DIF-Adjusted Factor Scores **eTable 8.** CFA and MNLFA Results With Module as Moderator CFA **eTable 9.** Mixed Effects Model With ADOS Calibrated Severity Scores (CSS) as Outcome **eTable 10.** Mixed Effects Model Without Language Level as Predictor **eTable 11.** Mixed Effects Model With Site as a Random Effect **eTable 12.** False Positive and False Negative Rates for ADOS CSS and FS-MNLFA Scores **eTable 13.** Configural Invariance for HFL Group by Race and Ethnicity **eTable 14.** Moderated Nonlinear Factor Analysis (MNLFA) Final Model Parameter Estimation Results for Social Communication (SC) and Restricted and Repetitive Behaviors (RRB) Domains With Full Equations **eFigure 1.** ADOS Score Distribution by Item, Likelihood Group, Sex, and Age Bin **eFigure 2.** Receiver Operating Characteristic (ROC) Curves **eFigure 3.** CFA Model Fit Indices for Each Race and Ethnicity Group **eFigure 4.** Scatterplots Depicting the Association Between Factor Estimates When Correcting for Age and Sex (http://ETA.SC/ETA.RRB) Compared With Those That Only Control for Sex but Are Split by Module (ETA.MD1.SC, etc)

Supplement 2.
Data Sharing Statement
