# Supplementary material for: Sex-Related Measurement Bias in Autism Spectrum Disorder Symptoms in the Baby Siblings Research Consortium
Source: JAMA Netw Open. 2025 Aug 8;8(8):e2525887. doi: 10.1001/jamanetworkopen.2025.25887 (PMC12334962; doi:10.1001/jamanetworkopen.2025.25887)
Supplement: Supplement 1. — eMethods. Participants, Measures, and Analytic Approaches eResults. MNLFA Results eTable 1. Demographic Characterization of Sample eTable 2. Mullen Early Learning Composite (ELC) Scores by Group eTable 3. Baby Sibling Research Consortium (BSRC) Site List With Principal Investigator (PI) eTable 4. ADOS Version and Module Administered eTable 5. Multigroup Confirmatory Factor Analysis (CFA) Configural Invariance by Sex and Age for HFL and LFL Participants eTable 6. Confirmatory Factor Analysis (CFA) Results for HFL-Negative Participants eTable 7. Descriptive Statistics of the MNLFA-Derived DIF-Adjusted Factor Scores eTable 8. CFA and MNLFA Results With Module as Moderator CFA eTable 9. Mixed Effects Model With ADOS Calibrated Severity Scores (CSS) as Outcome eTable 10. Mixed Effects Model Without Language Level as Predictor eTable 11. Mixed Effects Model With Site as a Random Effect eTable 12. False Positive and False Negative Rates for ADOS CSS and FS-MNLFA Scores eTable 13. Configural Invariance for HFL Group by Race and Ethnicity eTable 14. Moderated Nonlinear Factor Analysis (MNLFA) Final Model Parameter Estimation Results for Social Communication (SC) and Restricted and Repetitive Behaviors (RRB) Domains With Full Equations eFigure 1. ADOS Score Distribution by Item, Likelihood Group, Sex, and Age Bin eFigure 2. Receiver Operating Characteristic (ROC) Curves eFigure 3. CFA Model Fit Indices for Each Race and Ethnicity Group eFigure 4. Scatterplots Depicting the Association Between Factor Estimates When Correcting for Age and Sex (http://ETA.SC/ETA.RRB) Compared With Those That Only Control for Sex but Are Split by Module (ETA.MD1.SC, etc) [file jamanetwopen-e2525887-s001.pdf]

Supplementary Online Content

Burrows CA, Sung S, Zheng S, et al. Sex-related measurement bias in autism spectrum disorder symptoms in the Baby Siblings Research Consortium Study. *JAMA Netw Open.* 2025;8(8):e2525887. doi:10.1001/jamanetworkopen.2025.25887

eMethods. Participants, Measures, and Analytic Approaches
eResults. MNLFA Results
eTable 1. Demographic Characterization of Sample
eTable 2. Mullen Early Learning Composite (ELC) Scores by Group
eTable 3. Baby Sibling Research Consortium (BSRC) Site List With Principal Investigator (PI)
eTable 4. ADOS Version and Module Administered
eTable 5. Multigroup Confirmatory Factor Analysis (CFA) Configural Invariance by Sex and Age for HFL and LFL Participants
eTable 6. Confirmatory Factor Analysis (CFA) Results for HFL-Negative Participants
eTable 7. Descriptive Statistics of the MNLFA-Derived DIF-Adjusted Factor Scores
eTable 8. CFA and MNLFA Results With Module as Moderator
CFA
eTable 9. Mixed Effects Model With ADOS Calibrated Severity Scores (CSS) as Outcome
eTable 10. Mixed Effects Model Without Language Level as Predictor
eTable 11. Mixed Effects Model With Site as a Random Effect
eTable 12. False Positive and False Negative Rates for ADOS CSS and FS-MNLFA Scores
eTable 13. Configural Invariance for HFL Group by Race and Ethnicity
eTable 14. Moderated Nonlinear Factor Analysis (MNLFA) Final Model Parameter Estimation Results for Social Communication (SC) and Restricted and Repetitive Behaviors (RRB) Domains With Full Equations
eFigure 1. ADOS Score Distribution by Item, Likelihood Group, Sex, and Age Bin
eFigure 2. Receiver Operating Characteristic (ROC) Curves
eFigure 3. CFA Model Fit Indices for Each Race and Ethnicity Group
eFigure 4. Scatterplots Depicting the Association Between Factor Estimates When Correcting for Age and Sex (ETA.SC/ETA.RRB) Compared with Those That Only Control for Sex but Are Split by Module (ETA.MD1.SC, etc)

This supplementary material has been provided by the authors to give readers additional information about their work.

© 2025 Burrows CA et al. *JAMA Network Open*.

eMethods. Participants, Measures, and Analytic Approaches

Participants

Low-familial likelihood (LFL) participants who received an ASD diagnosis were not included in the analyses due to small sample size (*n*=24).

When multiple children from one family participated, we selected one child's data using the following prioritization criteria: female sex, most complete data, and random selection. When multiple data points were available in an age bin for a participant, one data point was randomly selected. Participants were removed when they were missing ADOS scores (n = 62). There were 1,196 (767 high-familial likelihood, HFL; 434 LFL) participants who did not receive clinical best estimate diagnosis of ASD at any visits.

Extended participant race characteristics are presented in eTable 1. Sites submitted information on race or ethnicity in text format and varied in their race/ethnicity categories. When sites submitted more than one category for race, participants were categorized into the "Mixed/Multiple" category. Examples of racial categories included in "Other" are: Bangladeshi, Caribbean, Brazilian, "Fil-Am", Kurdish, Mauritian, Mexican American, Sikh Indian, and Unknown/Not Reported/NA.

In terms of number of visits, 1,810 participants had one visit, 2,473 had two visits, and 267 had three visits.

Average Mullen Early Learning Composite (Mullen ELC) scores by age bin are presented in eTable 2.

Measures

Autism Diagnostic Observation Schedule (ADOS). Data collection for various sites occurred between 2003 and 2021; thus, both the ADOS-G (Lord et al., 2000) and ADOS-2 (Lord

et al., 2012) were used. ADOS version and module distribution by likelihood group, sex, and age bin is presented in eTable 4.

For the ADOS-2, Toddler Module, two codes assess hand/finger and complex mannerisms, as opposed to one code for other modules. We averaged participant scores across the two scores on the Toddler Module. The ADOS also has several codes with slightly different coding anchors for different modules (i.e., gestures, pointing, facial overtures directed to others). We combined codes across modules as we conceptualized these slight differences as reflecting age- and developmentally appropriate benchmarks for these behaviors.

Following ADOS scoring convention, scores of 3 were converted to 2. Scores of 7/8/9 were treated as missing. Missing data was coded as missing, not recoded to 0. ADOS score distributions for each code are depicted by group, sex, and age bin in eFigure1.

Masks and other personal protective equipment (PPE) were likely worn for visits between 2020 and 2021. BSRC data is not linked to date of visit, and we do not have information on whether or not masks were worn.

Mullen Scales of Early Learning (MSEL). The MSEL (Mullen, 1995) is a measure of cognitive and motor development standardized for children between birth and 68 months of age. Development is assessed through structured tasks assessing skills and abilities in 5 domains including gross motor, fine motor, visual reception, receptive language, and expressive language. The measure yielded subdomain *T*-scores and age-equivalent scores for each subdomain as well as an overall composite. The Mullen Early Learning Composite (ELC) score is presented in eTable2 to reflect overall developmental level of participants at various age bins and represents an aggregate score of all subdomains except for gross motor skills.

Analytic Approach

© 2025 Burrows CA et al. *JAMA Network Open*.

Moderated Nonlinear Factor Analysis (MNLFA). To evaluate differential item functioning (DIF) on latent factor loadings and item intercept across sex and age, we adopted the MNLFA approach, which is a highly flexible method, allowing to examine multiple, simultaneous moderators- either continuous or categorical. Sex was dummy coded (i.e., 0 = Female vs. 1 = Male) and age was entered as a continuous variable. For the MNLFA analyses, we employed an updated version of the *aMNLFA* package (v.1.1.2 ; Gottfredson et al., 2019) in R.

First, we evaluated model fit of each factor in each group and configural invariance across sex and age, using the CFA approach. Establishing good fit in each group and an overall common factor structure across groups (i.e., configural invariance) is a critical first step to test measurement invariance. Given that the multiple-group CFA approach only allows categorical moderators to test and cannot model multiple timepoints within individuals, age was discretized into three age groups, 20-26 months, 27-35 months, and 36-40 months of age. When participants had more than one visit within an age bin, we randomly selected one visit to use in the CFA. CFA analyses were conducted using the *lavaan* package (v.6-11; Rosseel, 2012) in R with WLSMV estimator for ordered categorical variables. Four model fit indices are reported: the χ^2 test, the comparative fit index (CFI), the root mean square error of approximation (RMSEA), and the standardized root mean square residual (SRMR). As the χ^2 test is almost always statistically significant for a model with large samples (400 or more), model fit was evaluated by other three fit indices. Values greater than .90 for the CFI, less than .08 for the RMSEA, and less than .08 for the SRMR were considered acceptable fit (Little, 2013).

For longitudinal data, MNLFA draws a calibration sample from independent observations to estimate the model parameters but uses the full longitudinal sample to estimate factor scores

© 2025 Burrows CA et al. *JAMA Network Open*.

combining all significant moderator effects. Thus, to test for model stability and ensure robustness of results, we conducted MNLFA analyses on five independent calibration samples and combined parameter estimates based on Rubin's rules (1987) to produce the final factor score estimation.

The model parameters were estimated through three steps. At the initial step, the effect of moderators on DIF for intercept and loading of each indicator was examined in a separate model, in the presence of effect of moderators on the latent factor. At the next step, all effects with $p < 0.05$ from the previous step were included and tested simultaneously in one model. Then, Benjamini-Hochberg family-wise error correction was applied to all model parameters to protect against type I errors. At the last step, a final model was estimated using the significant parameters ($p < 0.05$) after the Benjamini-Hochberg correction. Although the *aMNLFA* package implements automated *Mplus* codes, we reviewed and modified the automated *Mplus* codes at each estimation step to ensure that correct moderator effects are included. Items that showed significant DIF in any calibration sample were pulled into the final model, and the pooled effect was calculated and used in score estimation. This resulted in some final pooled estimates that were not statistically significant overall when correcting for multiple comparisons. Thus, we focus our discussion on items that showed final pooled estimates of $p \leq .01$.

Lastly, the resulting parameter estimates from the final model were then used to estimate the factor scores, which reflect not only individual's latent score but also the effects of significant moderators on DIF for intercept and loading of each indicator and latent factor mean and variance.

Sensitivity and Specificity. To evaluate the predictive utility of the FS_{mnlfa} in comparisons with raw ADOS scores, we conducted receiver operator characteristic (ROC) analyses[26] and the

© 2025 Burrows CA et al. *JAMA Network Open*.

two-sided DeLong test[27] to examine statistical differences of area under the curve (AUC) using two different scores. Using the optimal threshold values obtained by ROC analyses, we computed the rates of false-positive and false-negative using both the FS_{mnlfa} and the ADOS Calibrated Severity Score (CSS). The ROC analyses were conducted using the *pROC* packages (v1.18.5) in R.

The ROC analysis is typically displayed as a graph with the sensitivity of each possible cut score plotted on the y-axis and the specificity of each possible cut score plotted on the x-axis, illustrating how decision accuracy varies as the cut score is systematically moved across all possible values. We compared the graph's area under the curve (AUC), which quantifies overall decision accuracy. AUC values can range from 0.50 for decision accuracy at chance levels to 1.00 for perfectly accurate decisions. Following the guidelines by Jarrett et al. (2018) given the reliability and validity of psychological instruments, AUC values greater than 0.70 were considered clinically informative.

Results

MNLFA Results

The first step of MNLFA (Gotfredson et al., 2019) is to establish configural invariance across moderators of interest. Here, that involved examining confirmatory factor analysis (CFA) fit across HFL and LFL groups, at each age bin, and separately for males and females. CFA fit statistics are presented in eTable4 and eTable5. CFAs examining model fit by race and ethnicity are presented in eTable13 and eFigure3.

Due to differences in psychometric properties of the SC factor for HFL and LFL groups, we were interested in whether HFL-negative (non-ASD) siblings would show strong psychometric properties (similar to the HFL group overall), or whether there may be validity

concerns if we omitted the HFL-ASD group (similar to the LFL group results). Thus, we also examined CFA fit within HFL-negative participants at each age bin, and for males and females (eTable 5). Overall, fit statistics were strong for HFL-negative participants for both SC and RRB, indicating that the measurement models fit well in HFL non-cases.

In addition to the DIF tests presented in the main manuscript, the MNLFA model also examines overall mean level differences in factor score estimates by moderators. There was significant SC factor mean impact by sex (B = 0.93, SE = 0.19, $p < 0.001$) indicating that males had higher mean of latent SC factor. There was also a significant interaction effect of sex and age on SC factor mean (B = -0.02, SE = 0.007, $p = 0.005$) indicating that mean of latent SC factor decreased with time only in females. These results did not include the impact of ASD diagnosis, and thus we recommend referring to the mixed model results presented in the abstract.

For RRB, neither significant intercept DIF by sex nor mean impact by sex (B = 0.19, SE = 0.30, $p = 0.53$), age (B = 0.003, SE = 0.008, $p = 0.068$), or sex by age (B = 0.004, SE = 0.01, $p = 0.068$) were observed.

Due to the collinearity between age, language level, and ADOS module, we also ran the MNLFA using ADOS Module (1 vs. 2) as a covariate, rather than age. We only ran one calibration sample. Results were very similar to those that examined age as a moderator, and are presented in eTable8. A scatterplot of scores derived using age as a moderator and module as a moderator are presented in eFigure4.

Mixed effects with the language level

Including the language level as an additional predictor did not change the effects of sex, age, and ASD diagnosis SC_{mnlfa} and RRB_{mnlfa} (eTable 9, eTable 10). However, there was a significant main effect of the language levels and a significant interaction effect between the

© 2025 Burrows CA et al. *JAMA Network Open*.

language levels and ASD diagnostic status on SC_{mnlfa}. Children with higher language levels had significantly lower SC_{mnlfa} (B = -0.169, SE = 0.012, $p < 0.001$), controlling for sex, age, and ASD diagnostic status and the rate of decrease of SC_{mnlfa} as the language levels increases was greater in children with ASD diagnosis (B = -0.137, SE = 0.016, $p < 0.001$). By contrast, the language levels did not significantly predict RRB_{mnlfa}. However, there was significant interaction effect between the language levels and sex predicting RRB_{mnlfa} (B = -0.061, SE = 0.022, p = 0.007), indicating that the rate of decrease of RRB_{mnlfa} as the language levels increases was greater in males.

Sensitivity and specificity

Receiver operating characteristic (ROC) curves are presented in eFigure2. For SC, an AUC value using ADOS Social Affect algorithm scores was 0.922, 95% CI [.910 – 0.934] and an AUC value using SC_{mnlfa} was 0.940, 95% CI [.930 – 0.951]. The DeLong test revealed that SC_{mnlfa} had greater accuracy than ADOS Social Affect CSS, $z = 6.00$, $p < 0.001$. When examined by sex, AUC values were 0.937, 95% CI [.924 – 0.950] and 0.938, 95% CI [.919 – 0.957], using SC_{mnlfa} for males and females, respectively, and 0.924, 95% CI [.910 – 0.939] and 0.920, 95% CI [.898– 0.942] using ADOS Social Affect CSS for males and females, respectively. In both sexes, SC_{mnlfa} performed better than ADOS Social Affect CSS ($z = 3.30$, $p = 0.001$ and $z = 4.20$, $p <$ 0.001 for male and females, respectively).

For RRB, an AUC value using ADOS RRB algorithm scores was 0.868, 95% CI [.853 – 0.884] and an AUC value using RRB_{mnlfa} was 0.903, 95% CI [.889– 0.916]. The DeLong test revealed that RRB_{mnlfa} had greater accuracy than ADOS algorithm scores, z = 7.93, $p < 0.001$. When examined by sex, AUC values were 0.887, 95% CI [.870 – 0.905] and 0.909, 95% CI [.883 – 0.935], using RRB_{mnlfa}, for males and females, respectively, and 0.853, 95% CI [.833 –

© 2025 Burrows CA et al. *JAMA Network Open*.

0.872] and 0.873 95% CI [.843– 0.903] using ADOS algorithm scores for males and females, respectively. In both sexes, RRB_{mnlfa} performed better than ADOS RRB algorithm scores (z = 6.64, p <0.001 and z = 3.66, p = .00003 for male and females, respectively).

The optimal threshold values of ADOS CSS were 3.50 and 6.50 for SC and RRB, respectively. The optimal threshold values of ADOS algorithm scores were identical for males and females. The overall optimal threshold value of SC_{mnlfa} was 0.337 and 0.441 for males and females, respectively. The overall optimal threshold value of RRB_{mnlfa} was 0.464 and 0.746 for males and females, respectively. False positive and false negative rates using the optimal threshold values of ADOS algorithm scores versus FS_{mnlfa} are presented in eTable 10.

We also compared sensitivity and specificity of the adjusted factor scores (FS_{mnlfa}) to the ADOS CSS (thought to reflect routine clinical practice). When comparing the FS_{mnlfa} scores to ADOS Total CSS, the accuracy of SC_{mnlfa} did not significantly differ from the ADOS Total CSS (z = 0.11, p = 0.92) and RRB_{mlnfa} performed worse than the ADOS Total CSS (z = -5.21, p < .001).

© 2025 Burrows CA et al. *JAMA Network Open*.

eTable 1. Demographic Characterization of Sample

Characteristic	LFL<br>*n* = 1,444	HFL<br>*n* = 3,106
Race		
American Indian or Alaska Native	2 (0.13%)	7 (0.22%)
Asian	19 (1.31%)	136 (4.37%)
Black/African American	47 (3.25%)	70 (2.25%)
Native Hawaiian/Pacific Islander	0 (0%)	6 (0.19%)
White	746 (51.66%)	1751 (56.37%)
Mixed/Multiple	95 (6.57%)	272 (8.75%)
Other or Not Reported[a]	534 (36.98%)	864 (27.81%)

[a]*Examples of "other" for the race category include the following: Bangladeshi, Black/Caribbean, Brasilian , "Fil-Am", Kurdish, Mauritian, Mexican American, Sikh Indian, "Other", and "Unknown/not reported"*

© 2025 Burrows CA et al. *JAMA Network Open*.

eTable 2. Mullen Early Learning Composite (ELC) Scores by Group

Note: LFL = low-familial likelihood for ASD; HFL = high-familial likelihood for ASD. SD = standard deviation. Age is presented in months.

	LFL			HFL		
	n	ELC *Mean* (*SD*)	Age *Mean* (*SD*)	*n*	ELC *Mean* (*SD*)	Age *Mean* (*SD*)
20 – 26 months						
Overall	1157	111.48 (15.84)	24.23 (0.67)	2431	99.13 (19.50)	24.23 (0.80)
Female	538	115.74 (14.89)	24.27 (0.56)	1044	104.21 (18.64)	24.23 (0.80)
Male	619	107.90 (15.75)	24.20 (0.75)	1387	95.11 (19.23)	24.23 (0.79)
27 – 35 months						
Overall	199	112.09 (16.82)	28.00 (2.35)	704	96.53 (21.16)	28.28 (2.24)
Female	89	116.63 (15.47)	28.21 (2.32)	291	103.19 (20.53)	28.33 (2.28)
Male	110	107.88 (17.05)	27.79 (2.37)	413	91.45 (20.23)	28.25 (2.22)
36 – 40 months						
Overall	903	115.70 (15.85)	36.69 (1.07)	2163	101.89 (21.99)	36.85 (1.14)
Female	426	119.31 (15.08)	36.69 (1.05)	957	107.46 (20.06)	36.84 (1.10)
Male	477	112.39 (15.85)	36.69 (1.08)	1206	97.20 (22.44)	36.86 (1.17)

© 2025 Burrows CA et al. *JAMA Network Open*.

eTable 3. Baby Sibling Research Consortium (BSRC) Site List With Principal Investigator (PI)

Site	PI
Boston/Harvard University	Tager-Flusberg, Nelson
Calgary	Curtin
Canada	Zwaigenbaum
Emory University	Klin
Infant Brain Imaging Study Consortium	Piven
Kennedy Krieger Institute	Landa
University of Miami	Messinger
Pittsburgh	Iverson
University of South Carolina	Roberts
University of California - Davis	Hertz-Picciotto
University of California - Davis	Ozonoff
University of California - Los Angeles	Jeste
University of California - San Diego	Carver, Dobkins
United Kingdom/BASIS	Charman
University of Washington	Stone
University of Washington	Webb
Vanderbilt University	Stone
Yale University	Chawarska

© 2025 Burrows CA et al. *JAMA Network Open*.

eTable 4. ADOS Version and Module Administered

		ADOS-2	ADOS-G	Toddler Module	Module 1	Module 2
High Likelihood						
20 – 26 months	Female	245	799	171	799	74
	Male	352	1035	261	1079	47
27 – 35 months	Female	50	241	38	202	51
	Male	78	335	58	312	43
36 – 40 months	Female	268	689	0	181	774
	Male	368	838	0	362	840
Low Likelihood						
20 – 26 months	Female	143	395	99	369	70
	Male	192	427	127	437	55
27 – 35 months	Female	28	61	22	49	18
	Male	36	74	23	64	23
36 – 40 months	Female	106	320	0	15	405
	Male	145	332	2	39	427

© 2025 Burrows CA et al. *JAMA Network Open*.

eTable 5. Multigroup Confirmatory Factor Analysis (CFA) Configural Invariance by Sex and Age for HFL and LFL Participants

Domain	Moderator	χ^2	*df*	*p*	CFI	RMSEA	SRMR
High Likelihood							
Social Communication	Sex	476.123	70	< 0.001	0.951	0.062	0.032
	Age	849.942	105	< 0.001	0.950	0.064	0.032
Restricted and Repetitive Behavior	Sex	48.998	18	< 0.001	0.985	0.038	0.020
	Age	89.264	27	< 0.001	0.985	0.041	0.019
Low Likelihood							
Social Communication	Sex	216.766	70	< 0.001	0.870	0.054	0.041
	Age	303.061	105	< 0.001	0.888	0.051	0.039
Restricted and Repetitive Behavior	Sex	17.074	18	< 0.001	1.000	0.000	0.019
	Age	42.819	27	< 0.001	0.972	0.030	0.023

HFL Group

		χ^2	*df*	*p*	CFI	RMSEA	SRMR
Social Communication							
20 – 26 months	Female	96.467	35	< 0.001	0.958	0.072	0.039
	Male	201.375	35	< 0.001	0.93	0.102	0.042
27 – 35 months	Female	68.134	35	0.001	0.92	0.108	0.063
	Male	87.661	35	< 0.001	0.928	0.105	0.054
36 – 40 months	Female	160.489	35	< 0.001	0.922	0.115	0.056
	Male	151.589	35	< 0.001	0.927	0.114	0.045
Restricted and Repetitive Behavior							
20 – 26 months	Female	19.92	9	0.018	0.976	0.064	0.041
	Male	8.669	9	0.468	1	0	0.024
27 – 35 months	Female	4.084	9	0.906	1	0	0.026

© 2025 Burrows CA et al. *JAMA Network Open*.

		χ^2	*df*	*p*	CFI	RMSEA	SRMR
	Male	22.742	9	0.007	0.938	0.101	0.059
36 – 40 months	Female	14.84	9	0.095	0.988	0.058	0.029
	Male	28.077	9	0.001	0.977	0.07	0.032

LFL Group

		χ^2	*df*	*p*	CFI	RMSEA	SRMR
Social Communication							
20 – 26 months	Female[a]	*NA*	*NA*	*NA*	*NA*	*NA*	*NA*
	Male	89.794	35	< 0.001	0.845	0.093	0.077
27 – 35 months	Female[b]	*NA*	*NA*	*NA*	*NA*	*NA*	*NA*
	Male	52.695	35	0.028	0.872	0.137	0.103
36 – 40 months	Female[b]	*NA*	*NA*	*NA*	*NA*	*NA*	*NA*
	Male	55.913	35	0.014	0.755	0.187	0.086
Restricted and Repetitive Behavior							
20 – 26 months	Female	8.732	9	0.462	0.963	0.046	0.071
	Male	13.053	9	0.160	0.954	0.059	0.059
27 – 35 months	Female	11.226	9	0.261	0.808	0.182	0.107
	Male	9.835	9	0.364	0.968	0.082	0.090
36 – 40 months	Female	9.261	9	0.414	0.952	0.074	0.064
	Male	15.231	9	0.085	0.930	0.090	0.062

NA = not applicable, *a* = failed to run due to empty cell; *b* = failed to converge

© 2025 Burrows CA et al. *JAMA Network Open*.

eTable 6. Confirmatory Factor Analysis (CFA) Results for HFL-Negative Participants

		χ^2	*df*	*p*	CFI	RMSEA	SRMR
Social Communication							
20 – 26 months	Female	28.436	35	0.776	1.000	0.000	0.063
	Male	64.623	35	0.002	0.954	0.046	0.079
27 – 35 months	Female	50.62	35	0.043	0.925	0.092	0.239
	Male	26.264	35	0.857	1.000	0.000	0.148
36 – 40 months	Female	61.963	35	0.003	0.970	0.033	0.077
	Male	68.766	35	0.001	0.955	0.037	0.088
Restricted and Repetitive Behavior							
20 – 26 months	Female	6.191	9	0.721	1.000	0.000	0.055
	Male	5.504	9	0.788	1.000	0.000	0.048
27 – 35 months	Female	10.353	9	0.323	0.963	0.056	0.145
	Male	9.913	9	0.358	0.992	0.046	0.139
36 – 40 months	Female	7.712	9	0.563	1.000	0.000	0.040
	Male	9.878	9	0.360	0.998	0.012	0.042

© 2025 Burrows CA et al. *JAMA Network Open*.

eTable 8. CFA Results with Module as Moderator for high-familial likelihood (HFL) participants

	χ^2	*df*	*p*	CFI	RMSEA	SRMR
Social Communication						
Toddler Module	83.497	35	< 0.000	0.967	0.079	0.035
Module 1	445.511	35	< 0.000	0.934	0.101	0.04
Module 2	306.169	35	< 0.000	0.897	0.097	0.063
Configural Invariance by Module	496.035	105	< 0.000	0.992	0.039	0.048
Restricted and Repetitive Behavior						
Toddler Module	38.904	9	< 0.000	0.906	0.123	0.065
Module 1	29.576	9	0.001	0.99	0.048	0.023
Module 2	24.043	9	< 0.000	0.987	0.049	0.023
Configural Invariance by Module	81.666	27	< 0.000	0.986	0.037	0.018

© 2025 Burrows CA et al. *JAMA Network Open*.

MNLFA results in Module 1 with sex as a moderator

Domain	Parameter Type	Item	Estimate	SE	p^{a}
Social Communication	Loading	Unusual eye contact	0.573	0.022	< 0.001
		Facial expressions directed to others	0.368	0.016	< 0.001
		Showing	0.362	0.018	< 0.001
		Shared enjoyment in interaction	0.280	0.015	< 0.001
		Spontaneous initiation of joint attention	0.369	0.020	< 0.001
		Gestures	0.229	0.016	< 0.001
		Pointing	0.385	0.018	< 0.001
		Response to name	0.311	0.019	< 0.001
		Response to joint attention	0.221	0.015	< 0.001
		Quality of social overtures	0.437	0.015	< 0.001
	Intercept	Unusual eye contact	$0.542 + 0.142(Sex)$		
		Facial expressions directed to others	0.395	0.017	< 0.001
		Showing	0.678	0.019	< 0.001

© 2025 Burrows CA et al. *JAMA Network Open*.

		Shared enjoyment in interaction	0.228	0.015	< 0.001
		Spontaneous initiation of joint attention	0.451	0.021	< 0.001
		Gestures	0.435	0.016	< 0.001
		Pointing		$0.542 - 0.037(Sex)$	
		Response to name	0.409	0.024	< 0.001
		Response to joint attention	0.146	0.017	< 0.001
		Quality of social overtures		$0.401 + 0.058(Sex)$	
	Loading DIF	Item	Estimate	SE	*p*
		No items with significant loading DIF	-	-	-
	Intercept DIF	Unusual eye contact	0.142	0.038	< 0.001
		Pointing	-0.037	0.027	0.164
		Quality of social overtures	0.058	0.024	0.015
Restricted and Repetitive Behavior	Loading	Hand and finger and other complex mannerisms	0.366	0.018	< 0.001
		Immediate echolalia	0.269	0.019	< 0.001
		Intonation of vocalizations/verbalizations		$0.412 + 0.089(Sex)$	
		Stereotyped/idiosyncratic use of words/phrases	0.409	0.021	< 0.001

		Unusually repetitive interests/stereotyped behavior		$0.335 + 0.075(Sex)$	
		Unusual sensory interest in play material/person		$0.259 + 0.116(Sex)$	
	Intercept	Hand and finger and other complex mannerisms	0.423	0.019	< 0.001
		Immediate echolalia	0.481	0.017	< 0.001
		Intonation of vocalizations/verbalizations	0.310	0.019	< 0.001
		Stereotyped/idiosyncratic use of words/phrases	0.305	0.018	< 0.001
		Unusually repetitive interests/stereotyped behavior		$0.485 + 0.075(Sex)$	
		Unusual sensory interest in play material/person	0.293	0.016	< 0.001
	Loading DIF	Item	Estimate	SE	*p*
		Intonation of vocalizations/verbalizations	0.089	0.028	0.002
		Unusually repetitive interests/stereotyped behavior	0.087	0.033	0.010
		Unusual sensory interest in play material/person	0.116	0.030	< 0.001
	Intercept DIF	Unusually repetitive interests or stereotyped behavior	0.075	0.028	0.007

MNLFA results in Module 2 with sex as a moderator

Domain	Parameter Type	Item	Estimate	SE	*p*[a]
	Loading	Unusual eye contact	0.520	0.025	< 0.001
		Facial expressions directed to others	0.291	0.014	< 0.001

© 2025 Burrows CA et al. *JAMA Network Open*.

Social Communication		Showing		$0.176 + 0.060(Sex)$	
		Shared enjoyment in interaction	0.173	0.011	< 0.001
		Spontaneous initiation of joint attention	0.223	0.017	< 0.001
		Gestures	0.202	0.018	< 0.001
		Pointing	0.166	0.015	< 0.001
		Response to name	0.193	0.014	< 0.001
		Response to joint attention	0.043	0.006	< 0.001
		Quality of social overtures	0.336	0.016	< 0.001
	Intercept	Unusual eye contact	0.456	0.026	< 0.001
		Facial expressions directed to others	0.217	0.014	< 0.001
		Showing	0.346	0.015	< 0.001
		Shared enjoyment in interaction	0.096	0.010	< 0.001
		Spontaneous initiation of joint attention	0.261	0.016	< 0.001
		Gestures	0.603	0.017	< 0.001
		Pointing		$0.542 - 0.037(Sex)$	
		Response to name	0.197	0.014	< 0.001
		Response to joint attention	0.033	0.005	< 0.001
		Quality of social overtures	0.311	0.016	< 0.001
		Item	Estimate	SE	*p*
	Loading DIF	Showing	0.060	0.029	0.039
	Intercept DIF	Pointing	-0.059	0.027	0.026
Restricted and Repetitive Behavior	Loading	Hand and finger and other complex mannerisms	0.307	0.018	< 0.001
		Immediate echolalia	0.302	0.017	< 0.001
		Intonation of vocalizations/verbalizations	0.368	0.015	< 0.001
		Stereotyped/idiosyncratic use of words/phrases	0.353	0.014	< 0.001
		Unusually repetitive interests/stereotyped behavior	0.334	0.016	< 0.001

© 2025 Burrows CA et al. *JAMA Network Open*.

	Item	Estimate	SE	p
	Unusual sensory interest in play material/person		$0.188 + 0.077(Sex)$	
Intercept	Hand and finger and other complex mannerisms	0.335	0.019	< 0.001
	Immediate echolalia	0.349	0.018	< 0.001
	Intonation of vocalizations/verbalizations	0.271	0.018	< 0.001
	Stereotyped/idiosyncratic use of words/phrases	0.278	0.017	< 0.001
	Unusually repetitive interests/stereotyped behavior	0.327	0.022	< 0.001
	Unusual sensory interest in play material/person		$0.196 + 0.138(Sex)$	
	Item	Estimate	SE	p
Loading DIF	Unusual sensory interest in play material/person	0.077	0.028	0.006
Intercept DIF	Unusual sensory interest in play material/person	0.138	0.027	< 0.001

© 2025 Burrows CA et al. *JAMA Network Open*.

eTable 9. Mixed Effects Model with ADOS Calibrated Severity Scores (CSS) as Outcome

Domain	Model	Estimate	SD	*t*	*p*
Social Communication CSS	Fixed Effects				
	(Intercept)	2.338	0.151	15.522	< 0.001
	Age	0.003	0.005	0.529	0.597
	Sex	0.058	0.067	0.857	0.391
	ASD Dx	2.486	0.327	7.607	< 0.001
	Age × ASD Dx	0.035	0.011	3.233	0.001
	Random Effects				
	σ^2 (Intercept)	5.180			
	σ^2 (Age)	0.006			
	Corr (Age × Intercept)	-0.883			
	σ^2 (Residual)	1.896			
Restricted and Repetitive Behavior CSS	Fixed Effects				
	(Intercept)	3.889	0.273	14.259	< 0.001
	Age	-0.015	0.009	-1.801	0.072
	Sex	-0.261	0.363	-0.719	0.472
	ASD Dx	2.397	0.482	4.973	< 0.001
	Age × Sex	0.037	0.011	3.250	0.001
	Age × ASD Dx	0.044	0.014	3.104	0.002
	Sex × ASD Dx	-0.753	0.237	-3.180	0.002
	Random Effects				

© 2025 Burrows CA et al. *JAMA Network Open*.

σ^2 (Intercept)	15.666
σ^2 (Age)	0.013
Corr (Age × Intercept)	-0.922
σ^2 (Residual)	3.389

© 2025 Burrows CA et al. *JAMA Network Open*.

eTable 10. Mixed Effects Model Without Language Level as Predictor

Domain	Model	Estimate	SD	*t*	*p*
Social Communication	Fixed Effects				
	(Intercept)	0.202	0.048	4.253	< 0.000
	Age	-0.014	0.001	-9.752	< 0.000
	Sex	0.182	0.025	7.135	< 0.000
	ASD Dx	1.526	0.031	49.161	< 0.000
	Random Effects				
	σ^2 (Intercept)	1.515			
	σ^2 (Age)	0.001			
	Corr (Age × Intercept)	0.852			
	σ^2 (Residual)	0.200			
Restricted and Repetitive Behavior	Fixed Effects				
	(Intercept)	-0.083	0.070	-1.191	0.234
	Age	-0.002	0.002	-1.014	0.311
	Sex	0.055	0.093	0.596	0.552
	ASD Dx	0.791	0.121	6.509	< 0.000
	Age × Sex	0.008	0.003	2.655	0.008
	Age × ASD Dx	0.023	0.004	6.542	< 0.000
	Sex × ASD Dx	-0.308	0.067	-4.591	< 0.000
	Random Effects				
	σ^2 (Intercept)	0.776			
	σ^2 (Age)	0.001			

© 2025 Burrows CA et al. *JAMA Network Open*.

Corr (Age × Intercept)	0.726
σ^2 (Residual)	0.256

© 2025 Burrows CA et al. *JAMA Network Open*.

eTable 11. Mixed Effects Model With Site as a Random Effect

© 2025 Burrows CA et al. *JAMA Network Open*.

Domain	Model	Estimate	SE	95% CI [LL,UL]	*t*	*p*
Social Communication	Fixed Effects					
	(Intercept)	0.579	0.228	[0.131,1.027]	2.535	0.011
	Age	-0.004	0.008	[-0.019,0.012]	-0.446	0.655
	Sex	0.064	0.094	[-0.120,0.249]	0.685	0.493
	ASD Dx	1.528	0.126	[1.281,1.775]	12.136	< 0.001
	Language level	-0.188	0.013	[-0.273,-0.104]	-4.357	< 0.001
	Age × Sex	0.003	0.004	[-0.004,0.011]	0.874	0.382
	Age × ASD Dx	0.024	0.005	[0.015,0.033]	5.199	< 0.001
	Age × Language level	0.001	0.001	[-0.001,0.004]	1.064	0.287
	Sex × ASD Dx	-0.160	0.061	[-0.279,-0.040]	-2.617	0.009
	Sex × Language level	-0.001	0.020	[-0.041,0.039]	-0.035	0.972
	ASD Dx × Language level	-0.210	0.021	[-0.251,-0.169]	-9.992	< 0.001
	Random Effects					
	Level: Site					
	σ (Intercept)	0.299		[0.147,0.608]		
	σ (Slope)	0.008		[0.003,0.018]		
	Cor (Slope × Intercept)	-0.877		[-0.979,-0.421]		
	Level: ID					
	σ (Intercept)	1.117		[0.987,1.265]		
	σ (Slope)	0.030		[0.025,0.035]		
	Cor (Slope × Intercept)	-0.949		[-0.960,-0.936]		
	σ (Residual)	0.452				
	Fixed Effects					
	(Intercept)	-0.398	0.244	[-0.885, 0.080]	-1.632	0.103

© 2025 Burrows CA et al. *JAMA Network Open*.

Restricted and Repetitive Behavior	Age	0.017	0.009	[-0.000,0.034]	1.922	0.0548
	Sex	0.047	0.096	[-0.142,0.235]	0.483	0.629
	ASD Dx	0.842	0.130	[0.587,1.100]	6.480	< 0.001
	Language level	0.010	0.045	[-0.079,0.099]	0.216	0.829
	Age × Sex	0.016	0.004	[0.008,0.023]	3.932	< 0.001
	Age × ASD Dx	0.024	0.005	[0.014,0.033]	4.775	< 0.001
	Age × Language level	-0.002	0.001	[-0.005,0.001]	-1.485	0.138
	Sex × ASD Dx	-0.367	0.066	[-0.497,-0.237]	-5.543	< 0.001
	Sex × Language level	-0.052	0.022	[-0.095,-0.010]	-2.415	0.016
	ASD Dx × Language level	-0.034	0.023	[-0.079,0.010]	-1.517	0.129
	Random Effects					
	Level: Site					
	σ (Intercept)	0.315		[0.176,0.565]		
	σ (Slope)	0.010		[0.005,0.019]		
	Cor (Slope × Intercept)	-0.787		[-0.949,-0.292]		
	Level: ID					
	σ (Intercept)	0.774		[0.583,1.028]		
	σ (Slope)	0.023		[0.017,0.032]		
	Cor (Slope × Intercept)	-0.861		[-0.920,-0.763]		
	σ (Residual)	0.517				

© 2025 Burrows CA et al. *JAMA Network Open*.

eTable 12. False Positive and False Negative Rates for ADOS CSS and FS-MNLFA Scores

			ADOS CSS Score		MNLFA Factor Score	
			Negative	ASD	Negative	ASD
Social Communication						
	Total					
	ASD Dx	Negative	1663	385	1987	308
		ASD	60	530	68	603
		FNR	10.17%		10.13%	
		FPR		18.8%		13.42%
	Female					
	ASD Dx	Negative	803	189	982	129
		ASD	16	137	20	162
		FNR	10.46%		10.99%	
		FPR		19.95%		11.61%
	Male					
	ASD Dx	Negative	860	196	1005	179
		ASD	44	393	48	441
		FNR	10.07%		9.82%	
		FPR		18.56%		15.12%
			ADOS CSS Score		MNLFA Factor Score	
			Negative	ASD	Negative	ASD

© 2025 Burrows CA et al. *JAMA Network Open*.

Restricted and Repetitive Behavior	Total					
	ASD Dx	Negative	1964	293	2001	294
		ASD	186	456	151	520
		FNR	28.97%		22.50%	
		FPR		12.98%		12.81%
	Female					
	ASD Dx	Negative	992	103	987	124
		ASD	51	121	36	146
		FNR	29.65%		19.78%	
		FPR		9.41%		11.16%
	Male					
	ASD Dx	Negative	972	190	1014	170
		ASD	135	335	115	374
		FNR	28.72%		23.52%	
		FPR		16.35%		14.36%

Note. FNR= False Negative Rate, FPR = False Positive Rate

© 2025 Burrows CA et al. *JAMA Network Open*.

eTable 13. Configural Invariance for HFL Group by Race and Ethnicity

Domain	χ^2	*df*	*p*	CFI	RMSEA	SRMR
Social Communication	607.208	70	< 0.000	0.950	0.060	0.032
Restricted and Repetitive Behavior	83.282	18	< 0.000	0.979	0.045	0.021

© 2025 Burrows CA et al. *JAMA Network Open*.

eTable 14. Moderated Nonlinear Factor Analysis (MNLFA) Final Model Parameter Estimation Results for Social Communication (SC) and Restricted and Repetitive Behaviors (RRB) Domains with Full Equations

Domain	Parameter Type	Item	Source	Parameter Estimate	SE[a]	p[b]
Social Communication	Loading	Unusual eye contact		0.601	0.017	
		Facial expressions directed to others		$0.479 - 0.178(Sex) - 0.002(Age) + 0.006(SexAge)$ [c]		
		Showing		0.424	0.015	
		Shared enjoyment in interaction		0.308	0.009	
		Spontaneous initiation of joint attention		0.401	0.017	
		Gestures		0.236	0.015	
		Pointing		0.387	0.014	
		Response to name		0.352	0.017	
		Response to joint attention		$0.229 + 0.290(Sex) - 0.001(Age) - 0.008(SexAge)$		
		Quality of social overtures		$0.359 - 0.053(Sex) - 0.003(Age)$		
	Intercept	Unusual eye contact		$0.366 + 0.088(Sex) + 0.005(Age)$		
		Facial expressions directed to others		0.351	0.052	
		Showing		$0.764 - 0.007(Age)$		
		Shared enjoyment in interaction		$0.323 - 0.005(Age)$		
		Spontaneous initiation of joint attention		0.379	0.051	
		Gestures		$0.349 + 0.007(Age)$		
		Pointing		0.506	0.047	
		Response to name		$0.349 + 0.007(Age)$		
		Response to joint attention		0.117	0.030	
		Quality of social overtures		$0.349 + 0.007(Age)$		
	Loading DIF	Item	Source	Estimate	SE	p
		Response to joint attention	Sex	0.290	0.105	0.011
		Quality of social overtures	Sex	0.053	0.019	0.005
	Intercept DIF	Unusual eye contact	Sex	0.088	0.033	0.012

© 2025 Burrows CA et al. *JAMA Network Open*.

		Showing	Age	-0.007	0.002	0.003
		Shared enjoyment in interaction	Age	-0.005	0.001	0.004
		Gestures	Age	0.007	0.002	0.004
		Response to name	Age	-0.010	0.002	<0.001
	Mean Impact	η	Sex	0.930	0.190	<0.001
			Sex × Age	-0.010	0.007	0.005
	Variance	η	-	1.000	-	-
Restricted and Repetitive Behavior	Loading	Hand and finger and other complex mannerisms		0.344	0.015	
		Immediate echolalia		$-0.053 + 0.011(Age)$		
		Intonation of vocalizations/verbalizations		0.451	0.016	
		Stereotyped/idiosyncratic use of words/phrases		0.383	0.018	
		Unusually repetitive interests/stereotyped behavior		0.391	0.015	
		Unusual sensory interest in play material/person		$0.246 + 0.109(Sex)$		
	Intercept	Hand and finger and other complex mannerisms		0.357	0.082	
		Immediate echolalia		0.590	0.086	
		Intonation of vocalizations/verbalizations		$0.154 + 0.004(Age)$		
		Stereotyped/idiosyncratic use of words/phrases		0.263	0.093	
		Unusually repetitive interests/stereotyped behavior		$0.692 - 0.047(Sex) - 0.010(Age) + 0.006(SexAge)$		
		Unusual sensory interest in play material/person		0.218	0.063	
	Loading DIF	Item	Source	Estimate	SE	p
		Immediate echolalia	Age	0.011	0.003	0.005
		Unusual sensory interest in play material/person	Sex	0.109	0.026	< 0.001
	Intercept DIF	Unusually repetitive interests or stereotyped behavior	Age	-0.010	0.003	0.002
	Mean	η	-	0.000	-	-
	Variance	η	-	1.000	-	-

© 2025 Burrows CA et al. *JAMA Network Open*.

eFigure 1. ADOS Score Distribution by Item, Likelihood Group, Sex, and Age Bin

Note: LL = low-familial likelihood for ASD; HL = high-familial likelihood for ASD; M = male; F = female; ueye = Unusual Eye Contact; faceo = Facial Expressions Directed to Others; show = Showing; sijnt = Spontaneous initiation of Joint Attention; ges = Gestures; point = Pointing; rjnt = Response to Joint Attention; rname = Response to Name; shrnj = Shared Enjoyment in Interaction ; qsov = Quality of Social Overtures.

© 2025 Burrows CA et al. *JAMA Network Open*.

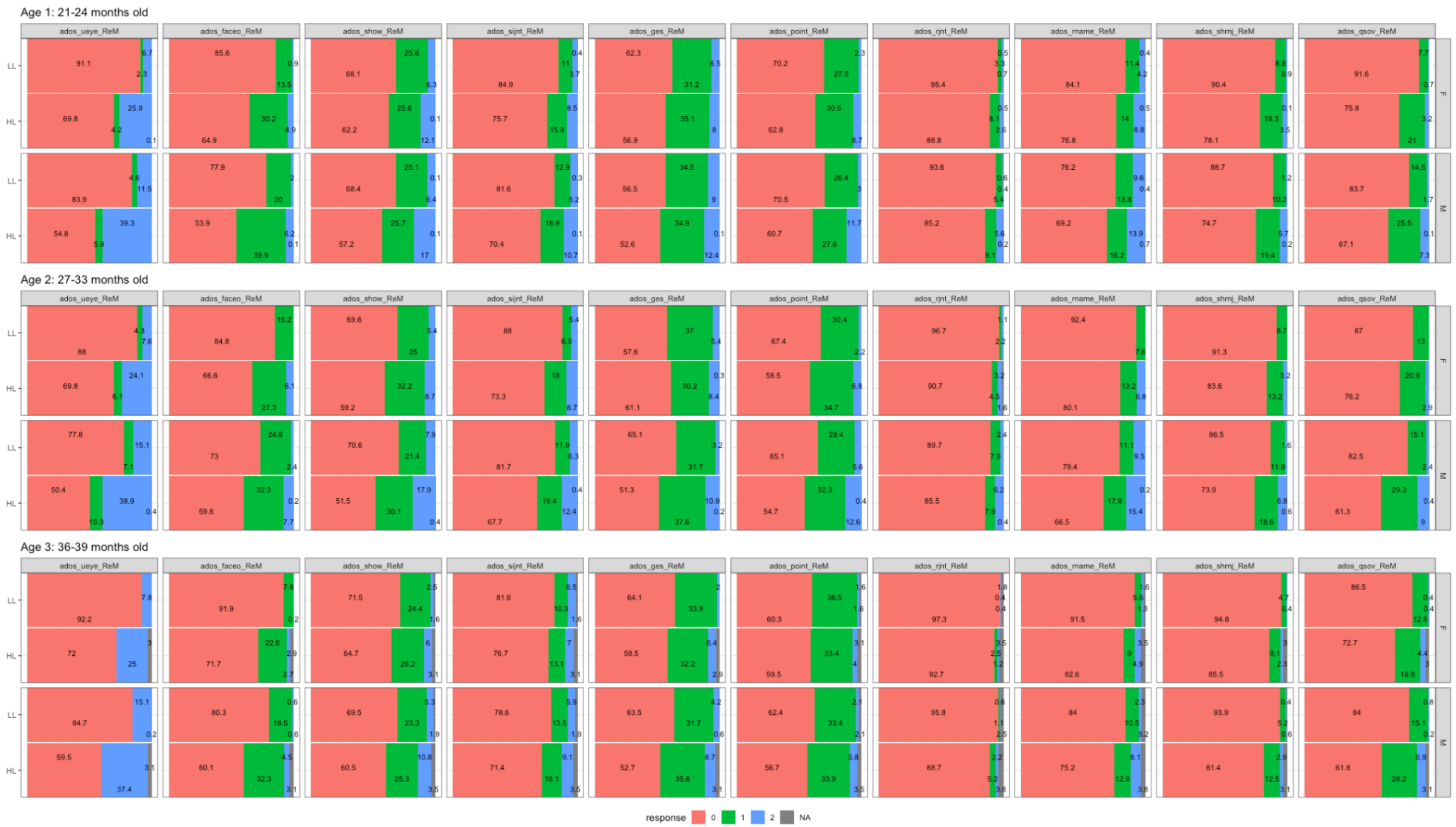

© 2025 Burrows CA et al. *JAMA Network Open*.

eFigure 2. Receiver Operating Characteristic (ROC) Curves

FS_{mnlfa} SC (A), ADOS SC domain score (B), FS_{mnlfa} RRB (C), and ADOS RRB domain score (D).

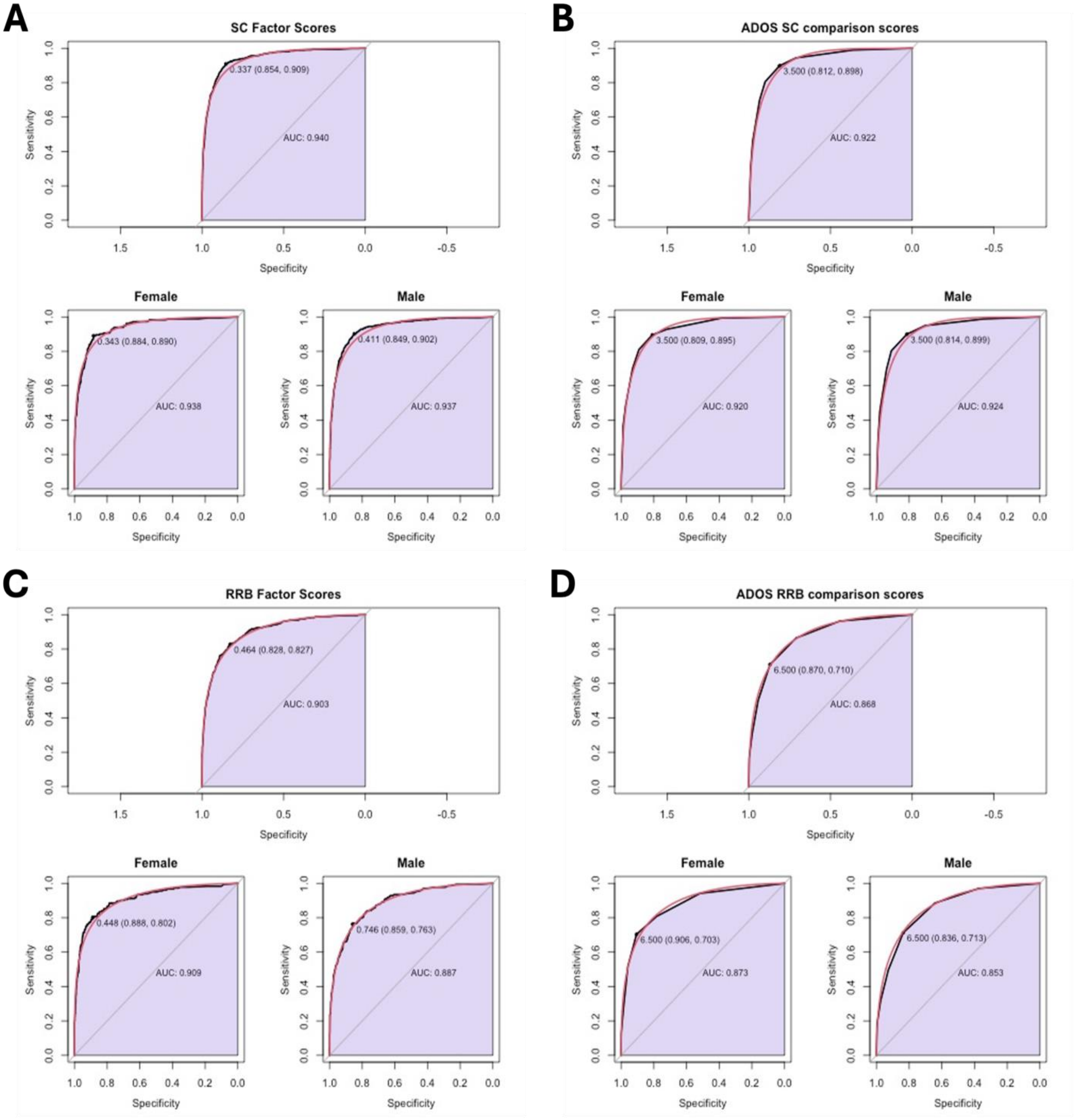

© 2025 Burrows CA et al. *JAMA Network Open*.

eFigure 3. CFA Model Fit Indices for Each Race and Ethnicity Group
social communication (A) and restricted and repetitive behavior (B) domains in the high-familial likelihood group.

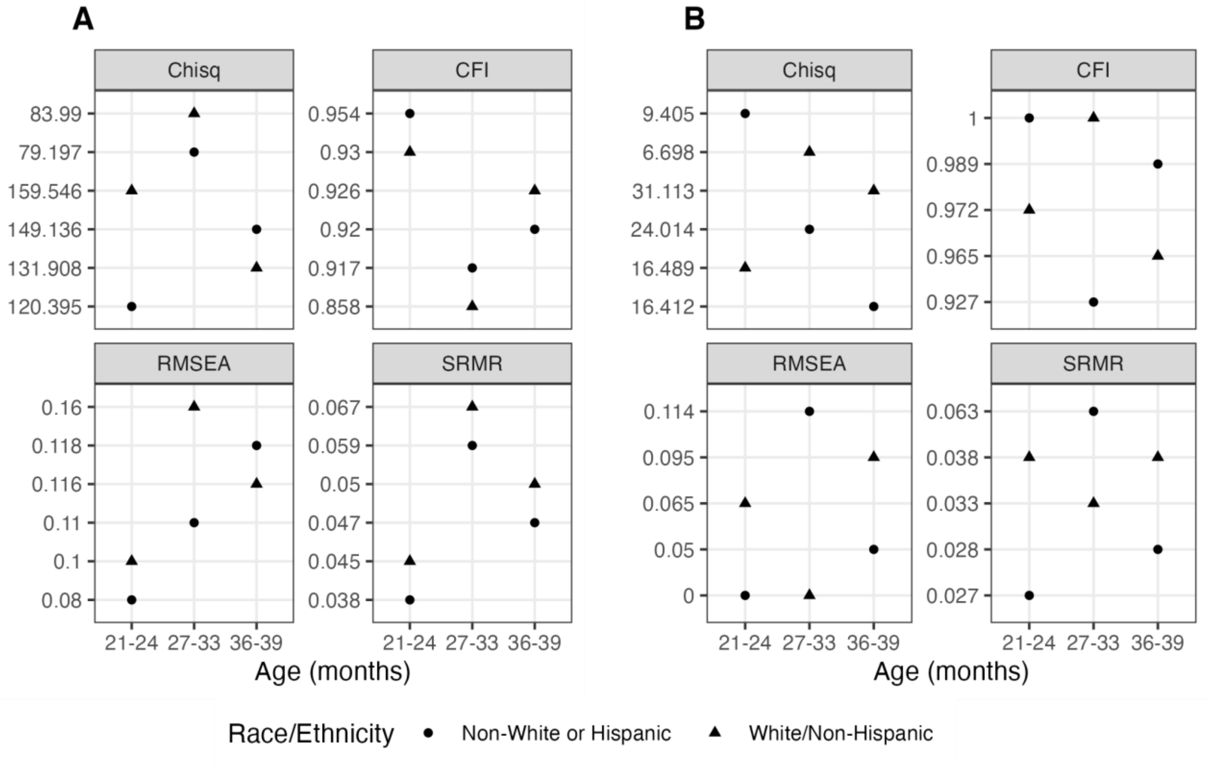

© 2025 Burrows CA et al. *JAMA Network Open*.

eFigure 4. Scatterplots Depicting the Association Between Factor Estimates When Correcting for Age and Sex (ETA.SC/ETA.RRB) Compared With Those That Only Control for Sex but Are Split by Module (ETA.MD1.SC, etc)

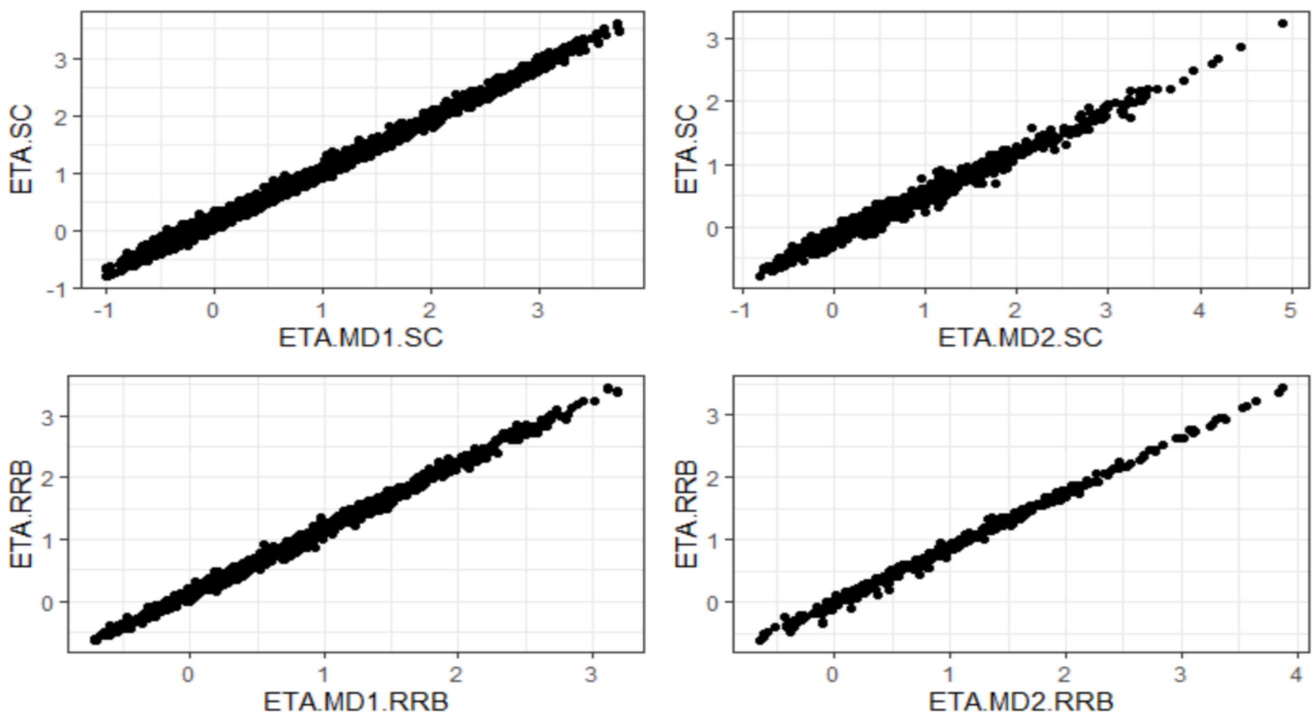

© 2025 Burrows CA et al. *JAMA Network Open*.
