# Supplementary material for: Sex-Related Measurement Bias in Autism Spectrum Disorder Symptoms in the Baby Siblings Research Consortium
Source: JAMA Netw Open. 2025 Aug 8;8(8):e2525887. doi: 10.1001/jamanetworkopen.2025.25887 (PMC12334962; doi:10.1001/jamanetworkopen.2025.25887)
Supplement: Supplement 2. — Data Sharing Statement [file jamanetwopen-e2525887-s002.pdf]

Data Sharing Statement

Burrows. Sex-Related Measurement Bias in Autism Spectrum Disorder Symptoms in the Baby Siblings Research Consortium Study. *JAMA Netw Open*. Published August 08, 2025. doi:10.1001/jamanetworkopen.2025.25887

Data
Data available: No

Additional Information
Explanation for why data not available: Data on common measures were deposited in a Baby Siblings Research Consortium (BSRC) Database by each site, providing an infrastructure for collaborative research projects. Data are submitted at least once a year. Access to the BSRC database can be made by collaboration with one of the BSRC members. Deidentified individual participant data will not be made available. CAB, SS and JTE had full access to all the data in the study and takes responsibility for the integrity of the data and the accuracy of the data analysis.
